# Exploring the Antimicrobial, Antioxidant and Extracellular Enzymatic Activities of Culturable Endophytic Fungi Isolated from the Leaves of *Kirkia acuminata* Oliv

**DOI:** 10.3390/microorganisms13030692

**Published:** 2025-03-19

**Authors:** Sagwadi Kubayi, Raymond Tshepiso Makola, Khumiso Dithebe

**Affiliations:** Department of Biochemistry, Microbiology and Biotechnology, University of Limpopo, Private Bag X 1106, Sovenga, Polokwane 0727, South Africa; 201808627@keyaka.ul.ac.za (S.K.); raymond.makola@ul.ac.za (R.T.M.)

**Keywords:** antimicrobial, antioxidant, anti-inflammatory, extracellular enzymes, fungal endophytes, secondary metabolites, South African medicinal plants

## Abstract

Fungal endophytes of medicinal plants produce diverse secondary metabolites and extracellular enzymes with therapeutic and biotechnological potential. However, the biological and biotechnological potential of fungal endophytes from South African medicinal plants remain relatively underexplored. In this study, the antimicrobial, antioxidant, anti-inflammatory and extracellular enzymatic capabilities of five fungal endophytes previously isolated from the leaves of *Kirkia acuminata* Oliv. were investigated. Sequencing of the internal transcribed spacer (ITS) regions revealed that the isolates belonged to the genera *Setosphaeria*, *Diaporthe* and *Corynespora*. The broth micro-dilution assay and the Folin–Ciocalteau reagent method were used to assess the antibacterial activity and the total phenolic content (TPC) of the fungal endophytes’ ethyl acetate crude extracts (CEs), respectively. The antioxidant activity was assessed using the ferric reducing antioxidant power (FRAP) and 1,1-diphenyl-2-picrylhydrazyl (DPPH) free radical scavenging assays. The influence of the CE of the *Setosphaeria rostrata* KaL-4 on the viability and LPS-induced interleukin-6 (IL-6) production in Raw 264.7 macrophages was assessed using a 3-(4,5-dimethylthiazol-2-yl)-2,5-diphenyltetrazolium bromide (MTT) assay and an ELISA, respectively. The ability of the isolates to produce extracellular proteases, laccases and peroxidases was also determined. The CEs displayed antimicrobial activity with MICs ranging from 0.63 to 1.25 mg/mL and reducing power and scavenging activity ranging from 40% to 18% and from 60% to 48%, respectively. The *S. rostrata* KaL-4 CE possessed the highest TPC and demonstrated dose-dependent cytotoxicity. The CE further demonstrated a significant reduction in IL-6 production at a concentration of 0.75 µg/mL. Only one isolate demonstrated the ability to produce proteases with an enzymatic index (EI) of 0.66, while laccases (EI range of 0.14 to 1.15) and peroxidases were produced by all of the isolates. These findings suggest that fungal endophytes from South African medicinal plants are promising sources of bioactive compounds and industry-significant extracellular enzymes.

## 1. Introduction

The emergence of drug-resistant pathogens is one of the major global health challenges. Several microbial pathogens have developed drug resistance mechanisms due to the misuse and/or overuse of antimicrobial drugs [1]. The increase in antimicrobial resistance (AMR) has diminished the number of antimicrobial agents which can be used to effectively treat and clear microbial infections. Microbial infections are a powerful trigger of the innate immune response and inflammation [2]. Prolonged microbial infections may possibly trigger hyper inflammation. While non-steroidal anti-inflammatory drugs (NSAIDs) are widely used to treat inflammation, the prolonged use of NSAIDs may lead to conditions such as renal injury, gastrointestinal toxicity, cardiovascular disorder and hepatoxicity [3], which affect their clinical application. This necessitates the search for novel and effective antimicrobial and anti-inflammatory agents with low toxicity.

Endophytes are microorganisms, mostly fungi and bacteria, which reside in healthy living plant tissue without causing any visible harm to the plant host [4]. The most widely studied group of endophytes, fungal endophytes, produce an array of structurally and chemically diverse secondary metabolites including those that are similar to their hosts [5]. The secondary metabolites produced by endophytic fungi belong to various classes of compounds, including terpenoids, alkaloids, flavonoids, quinones and phenols, which have been reported to possess various bioactivities including antimicrobial, anti-inflammatory, antioxidant and anticancer activities [6,7]. Fungal endophytes are not only excellent sources of secondary metabolites with therapeutic potential, but they also produce various extracellular enzymes [8].

Endophytic fungi use enzymes to penetrate plant tissue to facilitate colonization and obtain nutrition from the host as well as prevent the invasion of the host by phytopathogens [9]. These enzymes, which include cellulases, proteases, laccases, pectinases, lipases and peroxidases, can be applied in several industries, such as the pharmaceutical industry, as well as in food and wastewater treatment [10]. Their ability to withstand harsh conditions, such as temperature and pH, while producing little to no side reactions has made fungal enzymes to be the most sought-after product in industry [11]. Although fungal endophytes of medicinal plants have capabilities that can be utilized for the benefit of human health, the environment and industry, it is estimated that only 1% of fungal endophytes has been studied [12]. Additionally, the fungal endophytes of medicinal plants used in South Africa are relatively underexplored [13]. This leaves room for the exploration of medicinal plants used in South Africa as potential sources of novel fungal endophytes with the ability to produce important bioactive secondary metabolites and industry-significant extracellular enzymes.

The medicinal plant *Kirkia acuminata* Oliv., commonly known as White seringa, is widespread in tropical areas of sub-Saharan Africa where the fruits, leaves, roots and bark are used to treat abdominal pain, cough, snake bites, cholera and toothache in countries including South Africa [14,15]. This medicinal plant species is reported to be on the decline in the natural environment due to overharvesting by ethno-medicine practitioners looking to utilize the plant for its numerous medicinal uses [14]. Five fungal endophytes were previously isolated from the leaves of *K. acuminata* Oliv. [16]; however, the identity and the bioactivities and enzyme production potential of the isolates were not assessed.

The aim of this study was to investigate the antimicrobial, antioxidant, anti-inflammatory and extracellular enzymatic activities of the fungal endophytes isolated from *K. acuminata* Oliv. to assess their bioactive and biotechnological potential.

## 2. Materials and Methods

### 2.1. Identification of Endophytic Fungi

Five endophytic fungal isolates previously isolated from *Kirkia acuminata* Oliv. leaves [16] were plated on potato dextrose agar (PDA, Merck, Johannesburg, South Africa) and incubated for 7 days at 30 °C. The macromorphological characteristics of the cultures were observed to identify the different morphotypes. Molecular identification of the isolates was carried out at Inqaba Biotechnical Industry Ltd., Pretoria, South Africa. The Quick-DNA™ Fungal/Bacterial Miniprep Kit (Zymo Research, Catalogue No. D6005, Irvine, CA, USA) was used to extract genomic DNA from each isolate according to the manufacturer’s instructions. Polymerase chain reaction (PCR) was used to amplify the internal transcribed spacer (ITS) regions 1 and 2 with the forward ITS1 5′-TCCGTAGGTGAACCTGCGG-3′ and reverse ITS4 5′-TCCTCCGCTTATTGATATGC-3′ primers using the *OneTaq* mastermix (New England BioLabs Inc., Ipswich, MA, USA). The PCR products were visualized on 1% agarose gel (CSL-AG500, Cleaver Scientific Ltd., Warwickshire, UK) stained with EZ-vision^®^ Blue light DNA Dye. The PCR products were enzymatically purified using the ExoSAP procedure (NEB M0293L; NEB M0371; New England BioLabs Inc., Ipswich, MA, USA). The PCR products were purified using the ZR-96 DNA Sequencing Clean-up Kit™ (Zymo Research, Catalogue No. D4050, Irvine, CA, USA) and sequenced in the forward and reverse direction using the ABI 3730*xl* Genetic Analyzer (Applied Biosystems, Thermo Fisher Scientific, Waltham, MA, USA). The consensus sequence for each isolate was assembled using the CLC Bio Main Workbench, and the basic local alignment search tool (BLAST) analysis was performed on the National Center for Biotechnology Information (NCBI) website (https://blast.ncbi.nlm.nih.gov/Blast.cgi, accessed 15 September 2022) with default parameters [17] to match the sequences with those of known fungi in the GenBank database.

### 2.2. Fungal Cultivation and Extraction of Metabolites

To produce the secondary metabolites, the pure endophytic fungal isolates were first cultured on potato dextrose agar (PDA, Sigma-Aldrich, Johannesburg, South Africa) plates for 5–7 days at 30 °C. Thereafter, five mycelia plugs (5 mm diameter) of each actively growing culture were used to separately inoculate 500 mL Erlenmeyer flasks containing 200 mL potato dextrose broth (PDB, Sigma-Aldrich, Johannesburg, South Africa). The flasks were incubated at 25 °C for 15 days in the dark with periodical shaking at 150 rpm. Following the incubation period, the fermented broth was filtered using a Whatmann no. 1 filter (Merck, Johannesburg, South Africa) to remove fungal biomass, and the cell-free fermented broth was extracted three times with equal volumes of ethyl acetate in a separation funnel. The upper solvent layers containing the secondary metabolites were pooled together and then evaporated using a vacuum rotary evaporator (Hei-VAP Precision motor-lift; Heidoph, Schwabach, Germany) at 40 °C. The crude extracts (CEs) were re-suspended in dimethyl sulfoxide (DMSO; Sigma-Aldrich, Johannesburg, South Africa) to a final concentration of 10 mg/mL and stored at 4 °C.

### 2.3. Antibacterial Activity of Endophytic Fungal Crude Extracts

#### 2.3.1. Bacterial Test Organisms

The bacterial strains *Pseudomonas aeruginosa* (ATCC 25922), *Escherichia coli* (ATCC 27853) and *Klebsiella pneumonia* (ATCC 10031) were used as test cultures. The bacterial cultures were first plated on nutrient agar plates and incubated at 37 °C for 24 h.

#### 2.3.2. Minimum Inhibitory Concentration of Crude Extracts

The antibacterial activity of the endophytic fungal CEs was assessed to determine the minimum inhibitory concentration (MIC) of the CEs using the micro-broth dilution assay by Eloff [18] with minor modifications. Briefly, 100 µL nutrient broth was added to each well of a 96-well plate. Thereafter, 100 µL of the 10 mg/mL CEs were transferred to their respective wells and serially diluted by two-folds to achieve a concentration gradient of 2.5–0.02 mg/mL. Chloramphenicol (Merck, Johannesburg, South Africa) and DMSO were used as positive and negative controls, respectively. The 24 h old bacterial test cultures were suspended in nutrient broth to an OD_625_ value between 0.08 and 0.1 to match a 0.5 McFarland standard. Following this, 100 µL of each inoculum was transferred aseptically into each respective well, and the plates were incubated for 24 h at 37 °C. Following incubation, 40 µL of a 0.2 mg/mL *p*-iodonitrotetrazolium violet solution (INT, Sigma-Aldrich, Johannesburg, South Africa) was added, and the plates were incubated for 30 min at 37 °C. Viable bacterial cells are able to reduce the yellow INT to the pink-purple iodonitrotetrazolium formazan. The MIC of the CEs was taken as the lowest concentration at which no pink-purple colour change was observed.

### 2.4. Antioxidant Potential of Endophytic Fungal Crude Extracts

The antioxidant activity of the endophytic fungal CEs was determined using the ferric reducing antioxidant power (FRAP) and 1,1-diphenyl-2-picrylhydrazyl (DPPH; Merck, Johannesburg, South Africa) free radical scavenging assays. The CEs were diluted with sterile distilled water (dH_2_O) to a concentration of 2.5 mg/mL. All analyses were performed in triplicate, and the average values were used.

#### 2.4.1. Ferric Reducing Antioxidant Power (FRAP) Assay

The reducing potential of the endophytic fungal CEs was assessed using the method described by Arulpriya et al. [19]. Briefly, 100 µL of the CEs was mixed with 250 µL potassium ferricyanide (Sigma-Aldrich, Johannesburg, South Africa) and 250 µL phosphate buffer and incubated at 50 °C for 20 min. Thereafter, 250 µL trichloroacetic acid (Sigma-Aldrich, Johannesburg, South Africa) was added to the mixture and centrifuged at 3000 rpm for 10 min. Following this, 250 µL of the supernatant was mixed with 250 µL of dH_2_O and 50 µL of freshly prepared ferric chloride solution. The FRAP assay is a calorimetric assay that is dependent on the reduction of ferric iron (Fe^3+^) to ferrous iron (Fe^2+^) by electron-donating antioxidant compounds, resulting in the development of a dark blue colour. Absorbance was measured at 700 nm with a Multiskan FC plate reader (Thermo Scientific, Waltham, MA, USA). The ferric chloride and dH_2_O solution without CEs was used as a blank. Ascorbic acid (2.5 mg/mL; Sigma-Aldrich, South Africa) was used as a positive control.

Reducing power = (Average Absorbance of CE/Average absorbance of ascorbic acid) × 100(1)

#### 2.4.2. DPPH Free Radical Scavenging Assay

The free radical scavenging activity of the CEs was determined using a calorimetric DPPH assay as described by Baliyan et al. [20]. Briefly, 1 mL of the CEs (2.5 mg/mL) was mixed with 1 mL of DPPH [0.1 mM DPPH (Sigma-Aldrich, Johannesburg, South Africa) in 7.5 mL methanol (Rochelle Chemicals, Johannesburg, South Africa)]. The mixtures were shaken and incubated for 30 min in the dark at room temperature (RT). Following the incubation period, the absorbance of the mixture was measured at 517 nm using a Multiskan FC plate reader (Thermo Scientific, Waltham, MA, USA). Similarly, a 2.5 mg/mL ascorbic acid solution was used as a positive control, while water served as the blank. The redox reaction in this assay relied on purple-red-coloured free radical DPPH that turns yellow when reduced (scavenged). The antioxidants react with DPPH and reduce it to DPPH-H, and absorbance decreases. The degree of discoloration indicates the scavenging potential of the antioxidant compounds or extracts in terms of hydrogen donating ability. The scavenging activity of the CEs was illustrated as shown in the formula below:

% Scavenging activity = (Average Absorbance of CE/Average absorbance of ascorbic acid) × 100(2)

### 2.5. Total Phenolic Content of Endophytic Fungal Crude Extracts

The total phenolic content (TPC) of the CEs was determined using the Folin–Ciocalteau reagent (Sigma-Aldrich, Johannesburg, South Africa) method as described by Tambe and Bhambar [21]. A 10 µL volume of the CEs was added to distilled water to a final volume of 500 µL. Thereafter, 0.25 mL of the Folin–Ciocalteau reagent was added in each test tube, followed by the addition of 1.25 mL of sodium carbonate (Na_2_CO_3_, Merck, Johannesburg, South Africa). The mixtures were incubated for 30 min in the dark at RT. The absorbance of the mixtures was measured at 550 nm using the Genesys 10S ultraviolet/visible (UV-VIS) spectrophotometer (Thermo Scientific, Waltham, MA, USA). The same procedure was followed using varying concentrations of gallic acid (1.25–0.08 mg/mL; Merck, Johannesburg, South Africa) to construct a standard curve. The TPC was expressed as milligram gallic acid equivalent/gram of extract (mg GAE/g).

### 2.6. MTT (3-(4,5-Dimethylthiazol-2-yl)-2,5-diphenyltetrazolium) Assay

The effect of the *S. rostrata* KaL-4 CEs on the viability of Raw 264.7 macrophages was assessed using a 3-(4,5-dimethylthiazol-2-yl)-2,5-diphenyltetrazolium bromide tetrazolium salt (MTT; Thermo Scientific, Waltham, MA, USA) assay. This is a calorimetric assay that depends on the mitochondrial succinate dehydrogenase enzyme in viable cells [22]. The assay was initiated by seeding Raw 264.7 cells in a 96-well culture plate at a density of 3 × 10^5^ cells/100 µL for 3 h and allowed to adhere. Thereafter, the cells were treated with varying concentrations of CE (3 μg/mL, 1.5 μg/mL, 0.75 μg/mL, 0.375 μg/mL and 0.19 μg/mL) and 50 μg/mL curcumin for 24 h. This was followed by the addition of 5 mg/mL MTT to each well and further incubation for 1 h. Thereafter, the cell culture medium was aspirated, and the formazan was dissolved in 100 µL of DMSO and incubated for 10 min. Absorbance was then measured at 490 nm using a GloMax multiplex (Promega, Fitchburg, WI, USA). The data were plotted using GraphPad Prism 8 software (Sandiego, CA, USA).

### 2.7. Enzyme-Linked Immunosorbent Assay

The effect of the *S. rostrata* KaL-4 CE on the production of the pro-inflammatory cytokine, interleukin-6 (IL-6), in the lipopolysaccharide (LPS)-induced Raw 264.7 macrophages was assessed using an enzyme-linked immunosorbent assay (ELISA) using the method described in [23]. The Raw 264.7 cells were seeded in a 6-well plate at 3 × 10^5^ cells/mL for 3 h and allowed to adhere. Thereafter, the cells were stimulated with 10 μg/mL LPS for 3 h, treated with 0.75 μg/mL of the *S. rostrata* KaL-4 CE and further incubated for 24 h. Following treatment, the supernatant was harvested, and IL-6 production levels were measured using an ELISA according to the manufacturer’s guidelines (Elabscience Bionovation Inc., Houston, TX, USA). The IL-6 capture antibody was diluted with phosphate-buffered saline (PBS) to a concentration of 0.5 µg/mL. Thereafter, 100 µL of the antibody was added to each well and incubated overnight at RT. The unbound excess capture antibody was aspirated, and plates were washed 3× with 300 µL wash buffer (0.05% Tween-20 in 1× PBS). The standard was diluted by 2-fold, and 100 µL of diluted standard was added sequentially in triplicate. On the other hand, the samples were added in triplicate in separate wells and incubated for 2 h at room temperature. After 2 h of incubation, the supernatants were then aspirated, and the wells were washed 3× with wash buffer. The detection antibody was diluted in diluent to 0.5 µg/mL, and 100 µL of the detection antibody was added in each well for 2 h at RT. This was then followed by the aspiration of excess detection antibody, and plates were washed 3× with wash buffer. The 5.5 µL avian peroxidase was then diluted to a ratio of 1:2000; thereafter, 100 µL of this diluted avian peroxidase was added to each well and incubated for 30 min at RT. This solution was aspirated, and wells were washed 3× with wash buffer. Thereafter, 100 µL ABTS substrate solution was added to each well, the plates were incubated for colour development and 100 µL of the stop solution was added. Thereafter, the colour was measured by reading the absorbance at 405 nm with GloMax multiplex (Promega, Fitchburg, WI, USA). The data were plotted using GraphPad Prism 8 software (Sandiego, CA, USA).

### 2.8. Extracellular Enzymatic Activity of Fungal Endophytes

Endophytic fungal isolates were plated on PDA and incubated at 30 °C for 5–7 days. Thereafter, 5 mm mycelia plugs were used to inoculate glucose yeast extract peptone (GYP) agar media (glucose—1 g/L; yeast extract—0.1 g/L; peptone—0.5 g/L; agar—16 g/L) supplemented with enzyme-specific substrates to determine the ability of the isolates to produce laccases and proteases according to Sunitha et al. [24]. The test tube method was used to determine peroxidase activity using hydrogen peroxide.

#### 2.8.1. Protease Activity

The protease activity was determined by inoculating GYP media supplemented with 1 g gelatin (Sigma-Aldrich, Johannesburg, South Africa) at pH 6. The plates were the incubated for 5–7 days at 30 °C. The plates were then flooded with saturated ammonium sulphate (Merck, Johannesburg, South Africa). The formation of opaque agar and the enhanced clear zone observed around the active colonies indicate the ability of the isolates to produce extracellular proteases [24].

#### 2.8.2. Laccase Activity

Laccase activity was determined using GYP agar containing 0.05 g 1-naphthol/L (Sigma-Aldrich, Johannesburg, South Africa) at pH 6. The plates were incubated for 5–7 days at 30 °C. Following incubation, the colourless media turns blue around the fungal colonies that produce extracellular laccases [25].

#### 2.8.3. Peroxidase Activity

The fungal isolates were tested for peroxidase enzyme activity using a 3% hydrogen peroxide (H_2_O_2_; Rochelle solutions, Johannesburg, South Africa) solution in a test tube [26]. A 5 mm mycelia plug of each fungal isolate was separately immersed in 2 mL of the H_2_O_2_. The ability of the isolates to produce peroxidases is indicated by fizzing or bubbling in the test tube.

#### 2.8.4. Evaluation of Protease and Laccase Production

The enzymatic index (EI) of proteases and laccases was calculated to determine the relative enzymatic activities of the isolates. The EI was calculated using the formula by Chai et al. [27]:

EI = (diameter of hydrolysis zone − colony diameter)/colony diameter(3)

### 2.9. Statistical Analysis

The data are presented as the mean of three replicates ± the standard deviation. The statistical significance of the data was analyzed using Student’s *t*-test, with *p* < 0.05 considered statistically significant.

## 3. Results

### 3.1. Morphological and Molecular Identification of Fugal Endophytes

**Figure 1:**
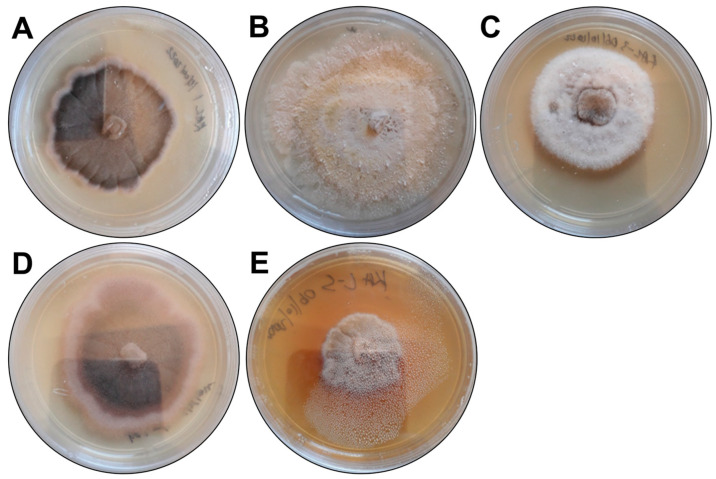
The morphological characteristics of the fungal endophytes growing on potato dextrose agar. The isolates were assigned the following codes: (**A**) KaL-1, (**B**) KaL-2, (**C**) KaL-3, (**D**) KaL-4 and (**E**) KaL-5.

The morphological characteristics of the endophytic fungal isolates were observed (Figure 1), and the fungal isolates were assigned the codes KaL-1 to KaL-5. ITS sequencing and the subsequent BLAST search results revealed that isolates KaL-1 and KaL-4 are both *Setosphaeria rostrata* and isolates KaL-2 and KaL-4 are both *Diaporthe* species, while KaS-3 is *Corynespora cassiicola* (Table 1).

### 3.2. Antibacterial Activity of Fungal Crude Extracts

The broth micro-dilution assay revealed that the CEs had varying inhibitory activity against the test pathogens, with MICs ranging from 0.63 to 1.25 mg/mL (Table 2). The CEs of the *S. rostrata* KaL-1, *Diaporthe* sp. KaL-2 and *Diaporthe* sp. KaL-5 isolates had high inhibitory activity against *P. aeruginosa* with an MIC of 0.63 mg/mL. Furthermore, the *Diaporthe* sp. KaL-5 isolate displayed high inhibitory activity against *E. coli* with an MIC of 0.63 mg/mL. All of the CEs had an MIC value of 1.25 mg/mL against *K. pneumoniae*.

### 3.3. Antioxidant Activity of Crude Extracts

**Figure 2:**
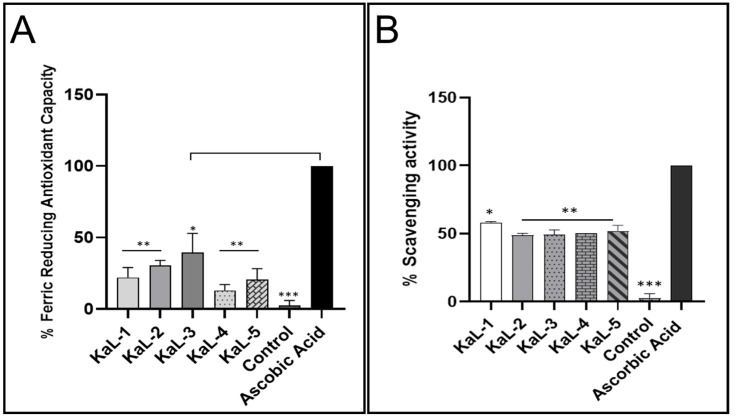
The antioxidant activity of the fungal endophyte crude extracts at 2.5 mg/mL. (**A**) The ferric reducing antioxidant capacity and (**B**) the free radical scavenging activity of the crude extracts. The data are presented as the mean of three replicates ± the standard deviation. The statistical analysis determines the significant difference between control and samples were *p* value is: * *p* < 0.05, ** *p* < 0.01, *** *p* < 0.001.

The antioxidant activity (Figure 2) of the endophytic fungal CEs was determined using the FRAP and the DPPH scavenging assays. All CEs were observed to have antioxidant activity using both the FRAP (Figure 2A) and DPPH (Figure 2B) assays. The reducing power ranged from as high as 40% to as low as 18%, while the radical scavenging potential ranged from 60% to 48%. The antioxidant activity for the different CEs was below ascorbic acid control for both assays. The *C. cassiicola* KaL-3 CE displayed the highest reducing potential of 40%, and the *S. rostrata* KaL-4 CE displayed the lowest reducing potential of 13%. The highest radical scavenging activity of 60% was observed for *Diaporthe* sp. KaL-5, while a radical scavenging activity of 48% was observed for *C. cassiicola* KaL-3, which was the lowest activity.

### 3.4. Total Phenolic Content of Fungal Endophyte Crude Extracts

The quantification of the TPC of the fungal endophyte CEs using the Folin-Ciocalteau reagent revealed that the CEs had varying levels of phenolic compounds (Table 3). The highest production of phenolic compounds was observed in the CE of the *S. rostrata* KaL-4 isolate with a TPC of 373.80 ± 12.39 mg GAE/g, while the CE of the *S. rostrata* KaL-1 isolate had the lowest TPC of 79.64 ± 2.36 mg GAE/g.

### 3.5. Influence of S. rostrata KaL-4 CE on Viability Profiles and IL-6 Production in RAW 264.7

**Figure 3:**
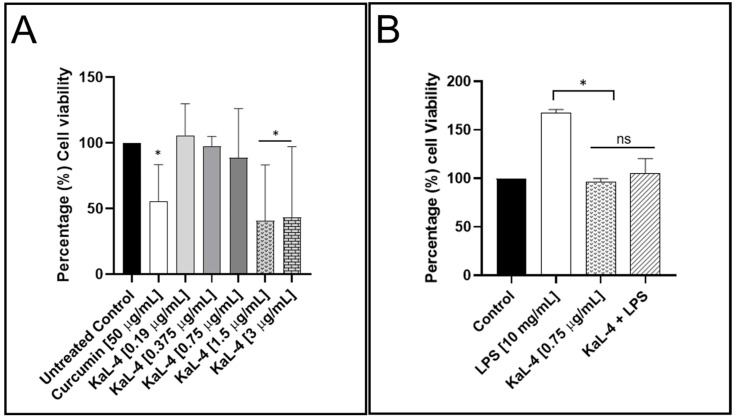
The effect of the *S. rostrata* KaL-4 crude extract on the viability and production of inflammatory mediators of Raw 264.7 macrophages. The crude extract displayed (**A**) dose-dependent cytotoxicity against the macrophages and (**B**) the ability to reduce interleukin-6 production (IL-6) in lipopolysaccharide (LPS)-induced Raw 246.7 cells at 0.75 µg/mL. The data are presented as the mean of three replicates ± the standard deviation. The statistical analysis determines the significant difference between control and samples were *p* value is: * *p* < 0.05. ns is non significant.

The effect of the *S. rostrata* KaL-4 CE on the viability profile of the RAW 264.7 macrophages and the production of the inflammatory mediator IL-6 was assessed using the MTT viability assay (Figure 3A) and ELISA (Figure 3B), respectively. The CE showed dose-dependent toxicity, with the viability of the cells being reduced to below 50% at high concentrations (1.5 and 3 µg/mL), while cell viability was observed to be above 90% at low doses (0.375 and 0.75 µg/mL). Interestingly, the CE was observed to enhance the viability of the macrophages at a concentration of 0.19 µg/mL. Moreover, the CE was observed to inhibit the production of IL-6 at the sub-lethal concentration of 0.75 µg/mL.

### 3.6. Extracellular Enzyme Production Capabilities of Fungal Endophytes

**Figure 4:**
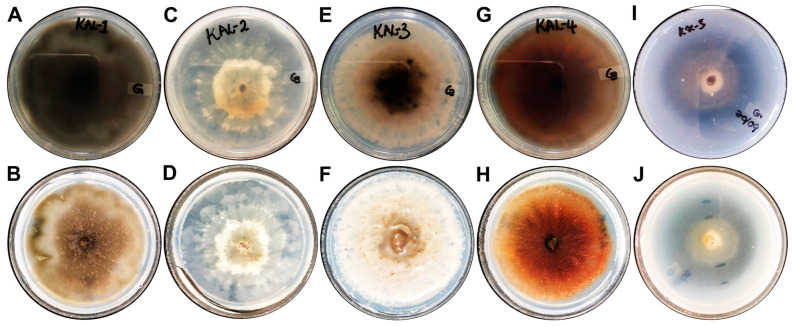
Extracellular protease activity for fungal endophytes. (**A**,**B**) *Setosphaeria rostrata* KaL-1, (**C**,**D**) *Diaporthe* sp. KaL-2, (**E**,**F**) *Corynespora cassiicola* KaL-3, (**G**,**H**) *Setosphaeria rostrata* KaL-4 and (**I**,**J**) *Diaporthe* sp. KaL-5.

**Figure 5:**
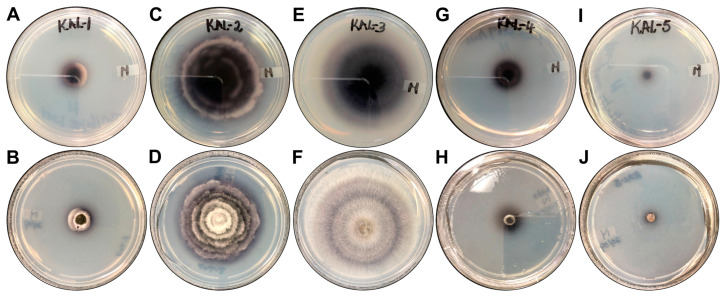
Extracellular laccase activity for fungal endophytes. (**A**,**B**) *Setosphaeria rostrata* KaL-1, (**C**,**D**) *Diaporthe* sp. KaL-2, (**E**,**F**) *Corynespora cassiicola* KaL-3, (**G**,**H**) *Setosphaeria rostrata* KaL-4 and (**I**,**J**) *Diaporthe* sp. KaL-5.

The extracellular protease and laccase activities of the fungal endophytes were determined on enzyme-specific solid media. The production of proteases and laccases varied amongst the isolates (Table 4). Only the *Diaporthe* sp. KaL-5 isolate displayed any protease activity (Figure 4) with an EI of 0.66 ± 0.05. The formation of a deep blue colour, indicative of laccase production, was observed around all colonies except the colony of the *C. cassiicola* KaL-3 isolate (Figure 5). However, it was observed that a deep blue colour had developed beneath the colony when the underside of the plate was inspected (Figure 5E,F). The highest laccase activity was observed for the *Diaporthe* sp. KaL-5 isolate with an EI of 1.15 ± 0.13, while an EI of 0.14 ± 0.01 made the *Diaporthe* sp. KaL-2 isolate the lowest producer of the laccase enzyme. The use of H_2_O_2_ to screen for peroxidase activity revealed that all isolates are able to produce extracellular peroxidases (Table 5).

## 4. Discussion

Medicinal plants are excellent sources of fungal endophytes with the ability to produce important bioactive secondary metabolites and extracellular enzymes. While numerous studies have focused on the biological activities of *K. acuminata* Oliv. leaves [28,29,30], there are no studies that have explored the fungal endophytes associated with the leaves of this medicinal plant species. In this study, the assessment of the macro-morphological features of the fungal endophyte colonies previously isolated from the leaves of *K. acuminata* Oliv. suggested the presence of five morphotypes. Macromorphological and microscopic features have been extensively used to categorize fungal endophytes as morphotypes and isolates; however, the use of macromorphological features to group fungal endophytes into morphotypes does not sufficiently represent the phylogeny of fungal endophytes, particularly for non-sporulating species [31]. This was made apparent by the fact that sequencing of the ITS region (ITS1-5.8S-ITS2) revealed that the five isolates represented three different genera with isolates KaL-1 and KaL-4 identified as *S. rostrata*, while isolates KaL-2 and KaL-5 were identified as *Diaporthe* species despite showing differences in their morphology. Despite the inability to delineate genera such as *Diaporthe,* the ITS region remains the standard DNA marker used for the identification of fungal endophytes [31], and it is important to couple the use of the ITS region to other DNA markers such as β-tubilin II and translation elongation factor 1α [32,33]. However, the selection of the alternative marker depends on the genus in question as the feasibility of the alternative marker cannot be readily transferred from one taxon to another, and an investigation is required for each taxon [33]. The molecular identification of the fungal endophytes revealed that all isolates used in this study belonged to the phylum Ascomycota. This coincides with other studies wherein the majority of fungal endophytes isolated from medicinal plants used in South Africa belonged to the phylum Ascomycota [32,34,35,36]. The low number of isolates obtained in this study correlates with the study by Abdalla et al. [35] wherein a total of five fungal endophytes were isolated from the leaves of three South African medicinal plants, namely, *Psychotria zombamontana* (Kuntze) Petit, *Catha edulis* (Vahl) Forssk. ex Endl. and *Melianthus comosus* Vahl. In contrast, Manganyi et al. [37] and Sishuba et al. [32] isolated 60 and 43 fungal endophytes, respectively, from the South African medicinal plant *Sceletium tortuosum* (L.). This demonstrates that the number of fungal endophytes obtained can vary from plant to plant.

Owing to the prevailing challenge of the increase in AMR, the secondary metabolites of fungal endophytes have been investigated for their antimicrobial potential [38,39]. In this study, the antibacterial activity of the CEs was tested against Gram-negative bacteria, which account for 90% of all urinary tract infections [40]. Gram-negative bacteria possess an LPS layer in their membranes, which makes them more resistant to antimicrobial agents than Gram-positive bacteria [41]. The *S. rostrata* KaL-1, the *Diaporthe* sp. KaL-2 and the *Diaporthe* sp. KaL-5 strains displayed high inhibitory activity against *P. aeruginosa* with an MIC of 0.63 mg/mL. Masoko [28] previously reported an MIC of 1.25 mg/mL for the leaf extracts of *K. acuminata* Oliv. against *P. aeruginosa*. Interestingly, Mabadahanye et al. [30] did not observe any inhibitory activity for the leaf crude extracts of *K. acuminata* Oliv. against *E. coli* using an agar well diffusion assay despite Masoko [28] reporting an MIC of 0.31 mg/mL for the DCM and methanol CEs. This indicates that the ethyl acetate CEs of fungal endophytes are more potent against *P. aeruginosa* and less potent against *E. coli* compared to the host plant CEs. Interestingly, the *S. rostrata* KaL-1 strain displayed higher efficacy against *P. aeruginosa* than the *S. rostrata* KaL-4 strain. Similarly, the *Diaporthe* species KaL-5 strain was more effective against *E. coli* than the *Diaporthe* sp. KaL-2 strain. These results suggest that the isolates may be different strains of the same species as their colony morphologies were also different. In contrast to the current study, Gagana et al. [42] reported that the ethyl acetate CEs of the *C. casiicola* isolate did not display any inhibitory activity against *E. coli*, *K. pneumoniae* and *P. aeruginosa* using the agar well diffusion method. Furthermore, the authors reported that the ethyl acetate CE of the *S. rostrata* isolate did not inhibit *E. coli* while displaying inhibitory activity against *K. pneumoniae* and *P. aeruginosa* [42]. Even though the MICs obtained in the study can be classified as moderate activity, Nxumalo et al. [43] highlighted that microbial extracts with MIC values lower than 1 mg/mL are considered to be noteworthy. As such, the CEs demonstrating an MIC of 0.63 mg/mL can be further explored for their potential as antimicrobial agents. Interestingly, Masoko [28] and Mabadahanye et al. [30] reported that the leaf extracts of *K. acuminata* Oliv. displayed inhibitory activity against *Staphylococcus aureus*. This warrants an investigation of the inhibitory activity of fungal endophyte CEs against Gram-positive bacteria as the CEs may potentially exhibit higher efficacy against Gram-positive bacteria than Gram-negative bacteria. Even though pathogenic bacteria produce biofilms that aid in conferring them with resistance against various antimicrobial agents [41], the CEs of fungal endophytes have been reported to possess anti-biofilm activity against biofilm-forming pathogens [44,45,46]. As such, the anti-biofilm activity of the CEs of the fungal endophytes used in this study needs to be investigated to fully understand the antimicrobial potential of the CEs obtained in the study.

The production of harmful free radicals has been linked with the development of various conditions, including cancer, neurodegenerative disease, diabetes and premature ageing [47]. Fungal endophytes have the ability to produce various secondary metabolites with antioxidant activities. Given that a single antioxidant assay is not adequate to measure the antioxidant efficacy of various crude extracts [48], the FRAP and the DPPH assays were used to assess the antioxidant activities of the CEs. Even though the fungal endophyte CEs displayed antioxidant activity, the reduction power and radical scavenging potential observed were below those of the ascorbic acid control. In general, CEs displayed higher radical scavenging activity than reducing potential. Our findings coincide with the findings by Pan et al. [49] wherein the ethyl acetate fractions of fungal endophytes isolated from the bulbs of *Fritillaria unibracteata* var. *wabuensis* were observed to generally display lower reducing power than radical scavenging activity. Antioxidants are known to possess a number of therapeutic properties, including antimicrobial, anti-inflammatory and anticancer properties [50]. Interestingly, the CE of the *C. cassiicola* KaL-3 strain displayed the highest antioxidant activity despite having an MIC value of 1.25 mg/mL against all tested pathogens.

The antioxidant activity of crude extracts is largely attributed to the presence of phenolic compounds [51]. Mabadahanye et al. [30] reported the presence of phenols in the acetone and methanol extracts but not in the ethyl acetate extracts of *K. acuminata* Oliv. leaves. In this study, phenols were detected in all ethyl acetate crude extracts albeit at varying concentrations. No significant difference in the TPC was observed for the CEs of *S. rostrata* KaL-1, *Diaporthe* sp. KaL-2 and *C. cassiicola* KaL-3 with a *p* value of less than 0.05. However, the CE of *S. rostrata* KaL-4 possessed a significantly higher TPC than all other isolates with a *p* value of less than 0.05. The significant difference in the TPC for the *S. rostrata* KaL-1 and KaL-4 strains suggests that they may be different strains of the same species. Similarly, the *Diaporthe* sp. KaL-5 strain displayed a significantly higher TPC than the *Diaporthe* sp. KaL-2 strain. This further suggests that the isolates may be different species altogether or different strains of the same species. The TPC recorded for the fungal endophytes in this study are higher than those reported by Ghazi-yaker et al. [52] and Yadav et al. [53], who recorded TPCs ranging from 6.4 to 125.32 mg/g GAE for fungal endophytes isolated from the leaves of *Ziziphus lotus* L. (Desf) and from 4.46 ± 0.15 to 60.13 ± 0.41 for fungal endophytes isolated from *Eugenia jambolana* Lam., respectively. Pan et al. [49] reported that the high antioxidant activity observed in the ethyl acetate crude extracts of fungal endophytes correlated with a high TPC. However, this was not the case in the current study as the CE of the *S. rostrata* KaL-4 strain displayed significantly lower reducing power than the other isolates despite having the highest TPC.

Inflammation is important in the clearance of infections; however, prolonged inflammation leads to oxidative stress, which has been linked with various ailments [54,55]. Secondary metabolites of fungal endophytes have been reported to reduce oxidative stress [56]. While the secondary metabolites of fungal endophytes are generally regarded as safe to use due to these microbes being symbionts of plants [57,58], the cytotoxicity of crude extracts on healthy cells needs to be investigated. The lack of studies on the safety of fungal endophyte crude extract may explain why little progress has been made in the development of commercial products from the secondary metabolites of fungal endophytes. Given that the CE of the *S. rostrata* KaL-4 strain possessed an overall high TPC, the influence of the CE on the viability of macrophages as well as the ability of the CE to modulate LPS-induced inflammation were assessed. The CE was not only non-cytotoxic and safe to use on normal macrophages at low doses, but it also attenuated the production of the pro-inflammatory cytokine IL-6 at a low concentration of 0.75 µg/mL. This demonstrates that only small doses of CE would be required to affect the requisite reduction in infection-induced inflammation. Recently, Koopklang et al. [59] reported that the endophytic *S. rostrata* SH8-8 strain isolated from *Ipomoea pes-caprae* produced a xanthone, ravelin, which demonstrated anti-inflammatory activity by suppressing nitric oxide production in LPS-induced macrophage J774A.1 cells. This demonstrates the potential for secondary metabolites from endophytic *S. rostrata* strains to be used for the treatment of inflammatory ailments. Although more work is required, the findings in this study suggest that the *S. rostrata* KaL-4 crude extract may interfere with the inflammatory signalling pathway that regulates the production of inflammatory mediators.

Microbial enzymes have been widely applied in various industries, including baking, food, biofuels and healthcare [60]. Endophytic fungi are well-documented sources of sought-after industry-significant extracellular enzymes with high stability and specificity [9]. In this study, the fungal endophytes were screened for their ability to produce proteases, laccases and peroxidases. Proteases are hydrolytic enzymes that hydrolyse peptide bonds of proteins and are a group of important enzymes that are broadly used in the food, pharmaceutical, leather and detergent industries and in bioremediation processes due to their high stability [60]. In this study, the enzymatic index (EI) of the fungal endophytic strains was determined according to Chai et al. [27] wherein isolates with EIs greater than 1.0 are considered to have strong enzyme production. In addition to being the only isolate to produce proteases, an EI of 0.66 ± 0.05 makes the *Diaporthe* sp. KaL-5 isolate an intermediate producer of enzymes. Given that the isolates KaL-2 and KaL-5 were identified as *Diaporthe* species, these results further suggest that they may be different strains or even different species of the same genus. All fungal endophyte isolates produced laccases, albeit at varying levels. Only the isolates *Diaporthe* sp. KaL-5 demonstrated strong laccase production with an EI of 1.15 ± 0.13, which was significantly higher (*p* < 0.05) than those of all other isolates. Furthermore, no significant difference was observed for the isolates *S. rostrata* KaL-1, *Diaporthe* sp. KaL-2 and *S. rostrata* KaL-4. While the EI of the *C. cassiicola* KaL-3 isolate could not be determined, it is important to note that the isolate exhibited the ability to produce the enzyme. Given that the glucose levels in the media may affect laccase production [61], it is possible that reducing the glucose content in the media may result in higher laccase activity for all isolates. Laccase is a dependent oxidative enzyme which has broad biotechnological applications, such as in industrial effluent detoxification, discoloration, the removal of phenolics from wine and pulp bleaching [10]. The production of laccases by the fungal endophyte *Diaporthe* sp. (syn. *Phomopsis* sp.) observed in this study correlates with the result obtained by Sunitha et al. [24] who observed extracellular laccase production by a *Phomopsis* species. Protease [62] and laccase [63] enzymes have also been reported to have antimicrobial activity, which highlights their significance in the pharmaceutical industry. All strains displayed the ability to produce peroxidases. Similarly, Attia et al. [26] observed that the majority of the fungal endophyte isolates from *Medicago sativa* demonstrated peroxidase activity. Peroxidase enzymes are important in industry for the breakdown of lignocellulosic biomass and the removal of dyes in textiles, and they are used as biosensors [64]. The results obtained in this study further highlight the potential of endophytic fungi to produce industry-significant enzymes.

## 5. Conclusions

The current study demonstrates that fungal endophytes from the leaves of *K. acuminata* Oliv. produce bioactive secondary metabolites with promising antimicrobial, antioxidant and anti-inflammatory properties. Thus, the full mycochemical composition of CEs should be determined to identify bioactive compounds. Furthermore, the physicochemical parameters required to enhance the yield and bioactivity of CEs should be investigated. In addition, the underlying molecular mechanisms of action of the CEs should be further explored. Although all fungal endophytes produce laccases and peroxidases, more research is required to understand the yield, stability and activities of the enzymes for economical, pharmaceutical, and industrial production. The findings in this study further highlight the potential of medicinal plant species used in South Africa as sources of endophytic fungi with important biological and biotechnological capabilities.

## Figures and Tables

**Table 1:** Colony morphology and molecular identification of fungal endophytes.

Isolate Code	Colony Description	Predicted Species	GenBank Best Match	
			Accession Number	% Identity
KaL-1	Grey irregular colony with black trim surrounded by white trim. Colony surface is raised and rough.	*Setosphaeria rostrata*	LT837843.1	100
KaL-2	Whitish-brown irregular colony with flat and rough surface.	*Diaporthe* species	MT355681.1	99.31
KaL-3	White circular colony with raised and soft surface.	*Corynespora cassiicola*	MK139711.1	100
KaL-4	Grey irregular colony surrounded by black, red and white trims, respectively. Colony surface is raised and rough.	*Setosphaeria rostrata*	LT837843.1	100
KaL-5	Whitish-brown irregular colony with raised and rough surface. Colour diffusing into medium.	*Diaporthe* species	MZ151534.1	99.65

**Table 2:** Minimum inhibitory concentrations (MICs) of fungal endophyte crude extracts (mg/mL).

Endophytic Fungi	*E. coli*	*K. pneumoniae*	*P. aeruginosa*
*Setosphaeria rostrata* KaL-1	1.25	1.25	0.63
*Diaporthe* sp. KaL-2	1.25	1.25	0.63
*Corynespora cassiicola* KaL-3	1.25	1.25	1.25
*Setosphaeria rostrata* KaL-4	1.25	1.25	1.25
*Diaporthe* sp. KaL-5	0.63	1.25	0.63
Chloramphenicol	0.02	0.02	0.02

**Table 3:** The total phenolic content (TPC) of the fungal endophyte crude extracts.

Endophytic Fungi	TPC (mg GAE/g)
*Setosphaeria rostrata* KaL-1	79.64 ± 2.36
*Diaporthe* sp. KaL-2	102.05 ± 1.77
*Corynespora cassiicola* KaL-3	143.97 ± 7.67
*Setosphaeria rostrata* KaL-4	373.80 ± 12.39
*Diaporthe* sp. KaL-5	231.42 ± 7.08

**Table 4:** The enzymatic index (EI) of fungal endophytes for the production of proteases and laccases. The data are presented as the mean of three replicates ± the standard deviation.

Endophytic Fungi	Protease Activity	Laccase Activity
*Setosphaeria rostrata*	−	0.43 ± 0.29
*Diaporthe* sp.	−	0.14 ± 0.01
*Corynespora cassiicola*	−	+
*Setosphaeria rostrata*	−	0.49 ± 0.02
*Diaporthe* sp.	0.66 ± 0.05	1.15 ± 0.13

−: negative; +: positive—the isolate displayed laccase activity.

**Table 5:** Peroxidase production ability of the fungal endophytes.

Endophytic Fungi	Peroxidase Activity
*Setosphaeria rostrata* KaL-1	+
*Diaporthe* sp. KaL-2	+
*Corynespora cassiicola* KaL-3	+
*Setosphaeria rostrata* KaL-4	+
*Diaporthe* sp. KaL-5	+

+: positive.

## Data Availability

The original contributions presented in this study are included in the article; further inquiries can be directed to the corresponding author.
